# Secondary neutron dose measurement for proton eye treatment using an eye snout with a borated neutron absorber

**DOI:** 10.1186/1748-717X-8-182

**Published:** 2013-07-17

**Authors:** Dong Wook Kim, Weon Kuu Chung, Jungwook Shin, Young Kyung Lim, Dongho Shin, Se Byeong Lee, Myongguen Yoon, Sung-Yong Park, Dong Oh Shin, Jung Keun Cho

**Affiliations:** 1 Department of Radiation Oncology, Kyung Hee University Hospital at Gandong, Seoul, Korea; 2 Radiation Oncology, University of California, San Francisco, USA; 3 Proton Therapy Center, National Cancer Center, Ilsan, Korea; 4 Department of Radiological Science, Korea University, Seoul, Korea; 5 Proton Therapy Center, McLaren Cancer Institute, Flint, MI, USA; 6 Department of Radiation Oncology, Kyung Hee University Medical Center, Seoul, Korea; 7 Department of Radiological Science, Jeonju University, Jeonju, Korea

**Keywords:** Proton, Secondary, Neutron, CR-39, Boron, Eye

## Abstract

**Background:**

We measured and assessed ways to reduce the secondary neutron dose from a system for proton eye treatment.

**Methods:**

Proton beams of 60.30 MeV were delivered through an eye-treatment snout in passive scattering mode. Allyl diglycol carbonate (CR-39) etch detectors were used to measure the neutron dose in the external field at 0.00, 1.64, and 6.00 cm depths in a water phantom. Secondary neutron doses were measured and compared between those with and without a high-hydrogen–boron-containing block. In addition, the neutron energy and vertices distribution were obtained by using a Geant4 Monte Carlo simulation.

**Results:**

The ratio of the maximum neutron dose equivalent to the proton absorbed dose (H(10)/D) at 2.00 cm from the beam field edge was 8.79 ± 1.28 mSv/Gy. The ratio of the neutron dose equivalent to the proton absorbed dose with and without a high hydrogen-boron containing block was 0.63 ± 0.06 to 1.15 ± 0.13 mSv/Gy at 2.00 cm from the edge of the field at depths of 0.00, 1.64, and 6.00 cm.

**Conclusions:**

We found that the out-of-field secondary neutron dose in proton eye treatment with an eye snout is relatively small, and it can be further reduced by installing a borated neutron absorbing material.

## Background

Proton beam therapy and heavy ion therapy are increasingly used because of their excellent dose localization performance. This high-precision localization is achieved by the Bragg peak effect, which affords a sharp distal fall-off in depth dose distribution compared with normal photon therapy. Proton therapy thus results in high target conformity and a very low integral dose delivered to the normal tissue. Although new photon therapy techniques such as intensity-modulated radiotherapy can enable dose distribution in a high-dose region comparable to that of proton therapy, the latter has the advantage of a low integral dose, which is related to low complication rates in normal tissue [1,2].

One area of great interest is the extra dose emitted by secondary neutrons produced by nuclear interactions with the material in the beam path during proton and heavy ion therapy. The amount of the secondary neutron dose generated from the proton or heavy ion therapy machines can be dependent on the beam delivery system because neutron production is highly dependent on the material in the beam path and on the design of the beam line. In addition, neutrons have high relative biological effectiveness (RBE) in that even a small dose of neutrons can have a large effect on the patient.

Neutron dose equivalents and neutron spectral fluences delivered outside the radiation field to patients undergoing proton treatment have been measured as a function of the lateral distance of the beam axis using a Bonner sphere and a LiI thermal neutron detector for particular scattered proton beams [3]. The neutron dose equivalent per proton absorbed dose in a large-field beam 50 cm from the isocenter has been estimated to range from 1.00 to 5.00 mSv/Gy [3]. For eye beams, this value was estimated to be about 0.02 mSv/Gy at the same distance. In 2009, the National Cancer Center (NCC) of Korea reported the secondary neutron doses for craniospinal irradiation (CSI), hepatocellular configuration (HCC), and prostate beam configuration for proton therapy using an allyl diglycol carbonate (CR-39) detector [4]. An early eye beam measurement [5] was also reported in 1998 that measured the secondary neutron dose inside an Alderson phantom for the Paul Scherrer Institute (PSI) proton spot beam with the maximum contribution (6.80 mGy) detected for a 60-Gy treatment dose. Similar studies have estimated secondary neutron doses from scattering and spot proton beam therapy [6-10].

Since 2007, the NCC has treated more than 400 patients with proton therapy using an Ion Beam Applications (IBA) PROTEUS 235 proton therapy machine. This proton system consists of a 230-MeV proton cyclotron, a beam line, two gantry rooms, and a fixed-beam room. The cyclotron provides a uniform 230 MeV of proton energy, and the energy is attenuated to a level appropriate for the treatment of the patient in the beam delivery system. For normal eye treatment, the energy degrader initially decreases the proton energy at the front of the beam line, and then, the beam delivery system in the treatment room ultimately attenuates the proton energy to ~60 MeV, which provides a range of approximately 3.00 cm. The fixed-beam room and the gantry room have similar systems inside except for the beam angle availability. These two types of treatment rooms are made applicable to eye treatment by attaching an eye snout, which provides a circular exit of diameter 4.00 cm for the beam field. The scattered eye proton beam loses its energy through its interaction with the material of the beam delivery system, including the eye snout, which may generate secondary neutrons. It is therefore important to understand and estimate the quantity of secondary neutrons produced by the eye beam delivery system.

We measured the secondary neutron dose equivalent per proton absorbed dose in a proton eye treatment unit in single scattering mode using a CR-39 neutron detector [10-16] at the NCC, Korea. We expected that the secondary neutrons from the beam delivery system could be reduced by using a neutron absorber before reaching the patient’s body. We therefore measured the secondary neutron dose equivalent per proton absorbed dose in a proton eye treatment unit in the single scattering mode with an additional high-hydrogen–boron-containing polyethylene block (SWX207HD5). Using the Geometry and Tracking (GEANT4, GEANT4 Collaboration) Monte Carlo (MC) method, we simulated the proton eye beam delivery system to determine the distribution of secondary neutron generation in the beam delivery system.

## Methods

### Neutron dose of the proton eye beam delivery system

In this study, a range of 3.23 cm and a spread-out Bragg peak (SOBP) of 3.17 cm for the proton beam were used to cover the target volume of an ocular melanoma or other eye malignancy with an eye snout [17-19]. The range was defined as the depth from the water surface to 90% of the peak position in distal fall-off. The SOBP was defined as the flatted output region from 90% of the peak position proximal to 90% of the peak position in the distal fall-off.

**Figure 1:**
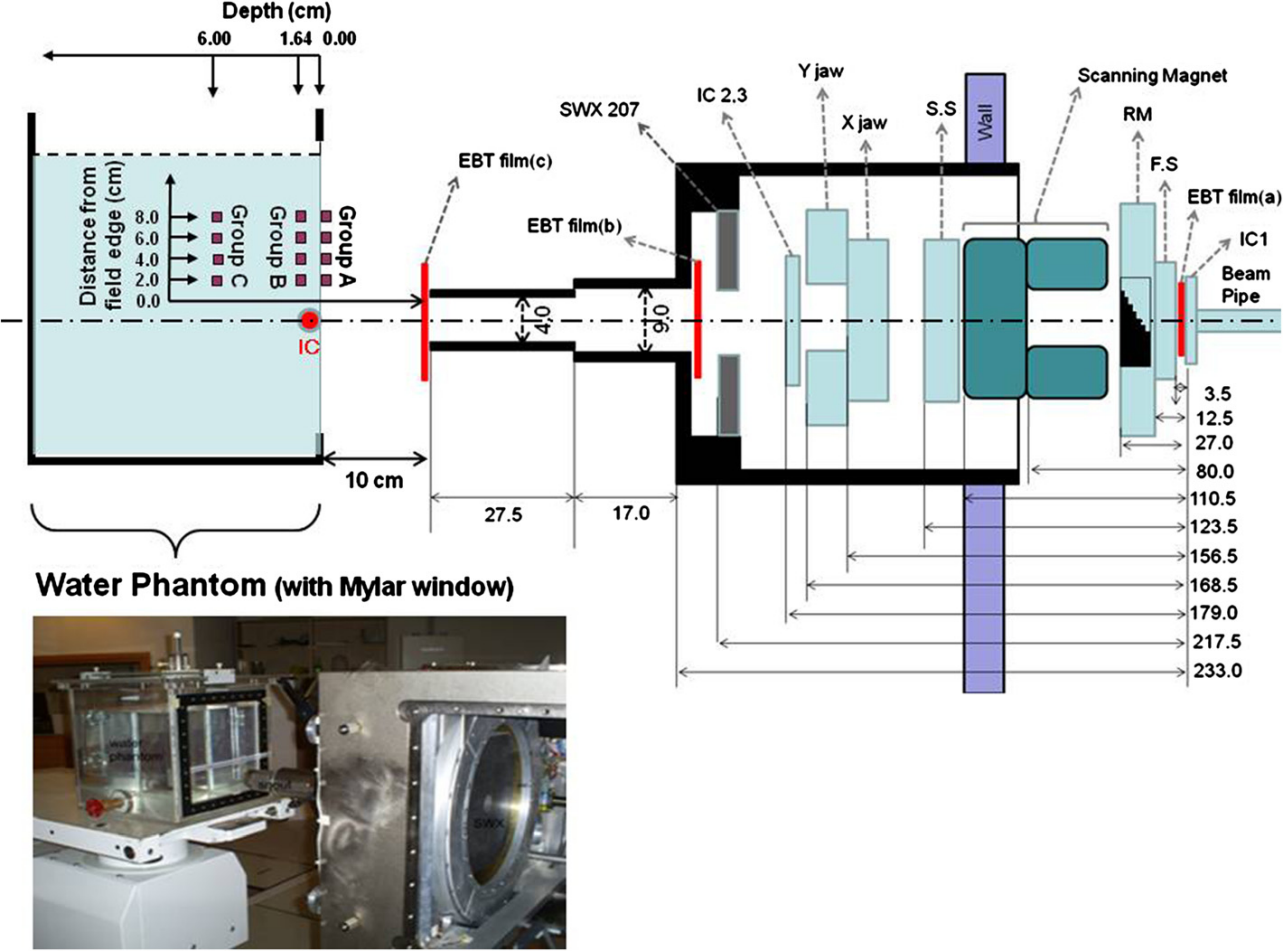
**Setup layout.** Diagram of the measurement set-up.

Figure 1 shows a side-view diagram of the measurement set-up. We used a 4.00-cm diameter for the proton beam field, which was created by a 4.00-cm-diameter circular, open brass block installed at the end of the eye snout. The air gap between the end of the eye snout and the water phantom was 10.00 cm. The proton beams entered the water phantom through a 0.0254-cm-thick Mylar window to reduce the proton range shift effect owing to the thickness of wall of the water phantom on the beam path.

The size of the eye beam field at the end of beam pipe was approximately 2.5 cm, and proton beam field size become broadened due to interactions with the materials in the beam delivery system such as the ion chambers, first scatter, and the range modulator (RM). The proton beam size between the ion chamber #3 and the front of the eye snout was measured to be 18.00 × 19.80 cm^2^ by using electron beam tomography (EBT) film with 10 × 10 cm^2^ of collimation. The difference between the *XY* collimator’s opening size and the measured field size is due to the distance between the film position and the collimator along the beam axis. In addition, the measured *X* and *Y* field sizes in the EBT film were different because the positions of the *X* and *Y* collimators along the beam axis are different. Include the *XY* collimator, most of the materials in the proton eye beam delivery system, can be a source of secondary neutrons by interaction with the protons. Finally, the field size was decreased to 4.00 cm in diameter by screening the eye snout materials and brass block, which potentially produces secondary neutrons by nuclear interaction.

**Figure 2:**
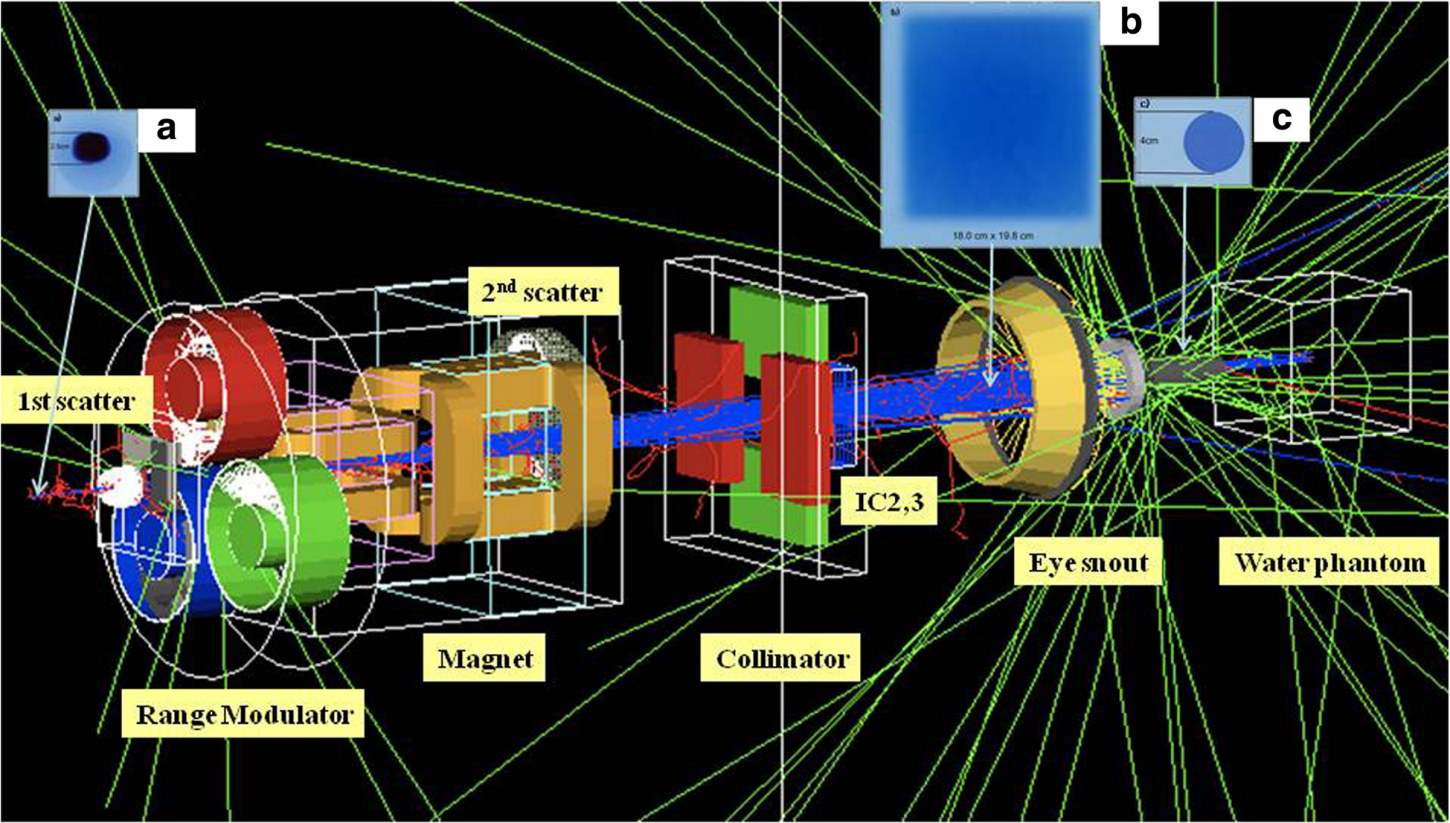
**Secondary neutron dose at proton eye snout.** View of the Geant4 simulation for the NCC proton eye beam delivery system. Most secondary neutrons were generated at the range modulator, the eye snout, and the water phantom. In the figure, three EBT film measurements at three different points are shown for the proton beam: **(a)** exit position of the beam pipe, **(b)** interim position between the eye snout and ion chamber 2, **(c)** exit position of eye snout. The proton beam was spread out in the lateral direction by interaction with materials in the beam path. We measured the lateral diameters of the proton beam at each position by using EBT film. From the exit of beam pipe to the front of the eye snout, the diameter of the beam cross section increased from **(a)** 2.5 cm to **(b)** 19 cm and then decreased to **(c)** 4 cm by the eye snout.

Figure 2 shows the EBT film measurements at each measuring position. The eye snout consists of brass, stainless steel, and other minor materials with brass being the dominant material.

CR-39 (Fukuvi Chemical Industry Co., Ltd., Fukui, Japan) was used to estimate the neutron dose produced by the eye beam delivery system in this study. The size of the sensitive area of the CR-39 detector was 46.8 mm^2^ in a 6.9-mm square. The thickness of the detector was 0.9 mm. The chemical composition of the CR-39 plastic track detector was C_12_H_18_O_7_ with a density 1.3 g/cm^3^. When particles pass through the CR-39 detector, they break the chemical bonds of the polymer and produce damage trails from recoil protons. The damage trails or tracks can be measured using an optical microscope after suitable chemical etching, electrochemical etching, or both. By counting a statistically significant number of tracks on the surface of the detector within a given area, the absorbed dose or dose equivalent value can be estimated.

The detectable energy with CR-39 ranges from 100 KeV to 20 MeV, which fully covers most of the secondary neutrons in proton therapy [15,16]. Therefore, the dose response of the relatively fast neutrons with energies of more than 20 MeV and the scattered neutrons with energies of less than 100 KeV are relatively low compared to that of relatively low neutrons with energies ranging from the 100 KeV to 20 MeV. The relatively fast neutrons are produced dominantly by the direct process at the position closer to the beam axis; therefore, the inefficiency of the detector can affect the measurement at near the field edge. However, we evaluated the relatively fast portion of the neutrons using the MC simulation. The relatively fast portion of the neutrons is less than 3% of the total neutron energy spectrum from the MC simulation study. The uncertainty due to the inefficiency of the CR-39 for scattered neutrons still exists in this study. In addition, primary protons, which have higher energy, will not produce tracks in the detector, because their energies are above the threshold of the detector.

To estimate the external neutron dose produced by the eye beam delivery system, CR-39 detectors were installed on the surface of the water phantom wall at distances of 2.00 cm, 4.00 cm, 6.00 cm, and 8.00 cm from the edge of the field (group A). To measure the neutron dose at the target and in normal tissue, CR-39 detectors were installed at depths of 1.64 cm and 6.00 cm at the same distances from the field edge (groups B and C, respectively).

### Neutron dose with SWX207HD5

To determine the change caused by the secondary neutron dose, a 3.00-cm-thick piece of SWX207HD5 self-extinguishing borated polyethylene (Shieldwrex™, Rio Rancho, New Mexico, USA) neutron shielding with a 4.00-cm hole was installed 70 cm from the isocenter. SWX207HD5 is composed of 5.50% boron and 5.70% hydrogen, and it has characteristics suitable for neutron attenuation. The macroscopic thermal neutron cross-section is 0.41 cm^−1^, and the gamma and neutron resistance is 5.0 × 10^6^ Gy and 2.5 × 10^17^ n/cm^2^, respectively. The recommended temperature limit is 93.30°C, and the solubility in water is negligible. In addition, this material is a better attenuation material for a proton beam compared with normal polymethyl-methacrylate (PMMA) because it has a higher density (1.60 g/cm^3^) than normal PMMA (1.18 g/cm^3^). We expected that a normal eye treatment beam, which has less than a 4.00-cm range, could be stopped by using a 3.00-cm-thick piece of SWX207HD5. CR-39 detectors were installed on the surface (group A), and at depths of 1.64 cm (group B) and 6.00 cm (group C) in the water phantom at various distances from the beam field edge.

### Neutron dose equivalent to the proton absorbed dose

The neutron dose equivalent to the proton absorbed dose was evaluated by comparing each CR-39 measurement and the output measurement of the 0.015 cc pinpoint chamber (type 31014 PTW: Freiburg, Germany) with a DOSE1 electrometer chamber (IBA Dosimetry: Schwarzenbruck, Germany) at the mid-depth of the SOBP (1.64 cm) in the water phantom. The CR-39 detectors were calibrated at the Korea Atomic Energy Research Institute with a D_2_O-moderated ^252^Cf neutron source. The calibration room is 8 m long, 6 m wide, and 6 m high. The source and detector were placed at a height of 2.9 m near the center of the room. The dose equivalent H was calculated for moderated and un-moderated neutron spectra based on the data provided in International Commission on Radiological Protection (ICRP) Publication 74. We used CR-39 detectors calibrated as H*(10) dose equivalents using ^252^Cf from the Korea Atomic Energy Research Institute. By cross-checking dose measurements between the independently calibrated CR-39 detectors and a ^3^He Swendi-2 neutron detector [15,20,21] located 100 cm from the isocenter in the vertical direction to the beam axis, we found that the measurements recorded by the two detectors were concordant within 14%. This indicates that the CR-39 detectors are useful dosimetry tools for measuring neutron dose. For cross-checking, we chose a 22-cm range with a 5-cm SOBP.

### Monte Carlo simulation

In 2010, the NCC [22] reported on the GEANT4 MC-based simulation for the IBA PROTEUS235 passive proton beam delivery system using a modulation wheel at the NCC. In that report, they introduced the fundamental class, which is an object for constructing the geometry and materials of the proton beam delivery system including the eye snout. The NCC presented the user physics list of the GEANT4 and the validation of a pristine Bragg peak and an SOBP in the report. By using the same tool, the proton eye beam delivery system was simulated as shown as Figure 2. To calibrate from the exit hall of the beam pipe to the end of the snout, 110 logical volumes were constructed and assigned to each component of the eye beam delivery system. The portion of secondary neutrons generated at each sub-part of the eye beam delivery system when secondary neutrons entered the 26 × 26 × 20 cm^3^ water phantom from the incident protons was calculated to find the major contribution to the neutron dose. To find the neutron-generated component in the eye beam delivery system, the vertex position and parent information of each neutron track that entered the water phantom were stored.

For this MC study, 1,152,112,300 neutron events and 1,022,850,920 proton events were generated and simulated for the proton eye beam delivery system with and without the SWX207HD5 block inside the eye snout.

**Figure 3:**
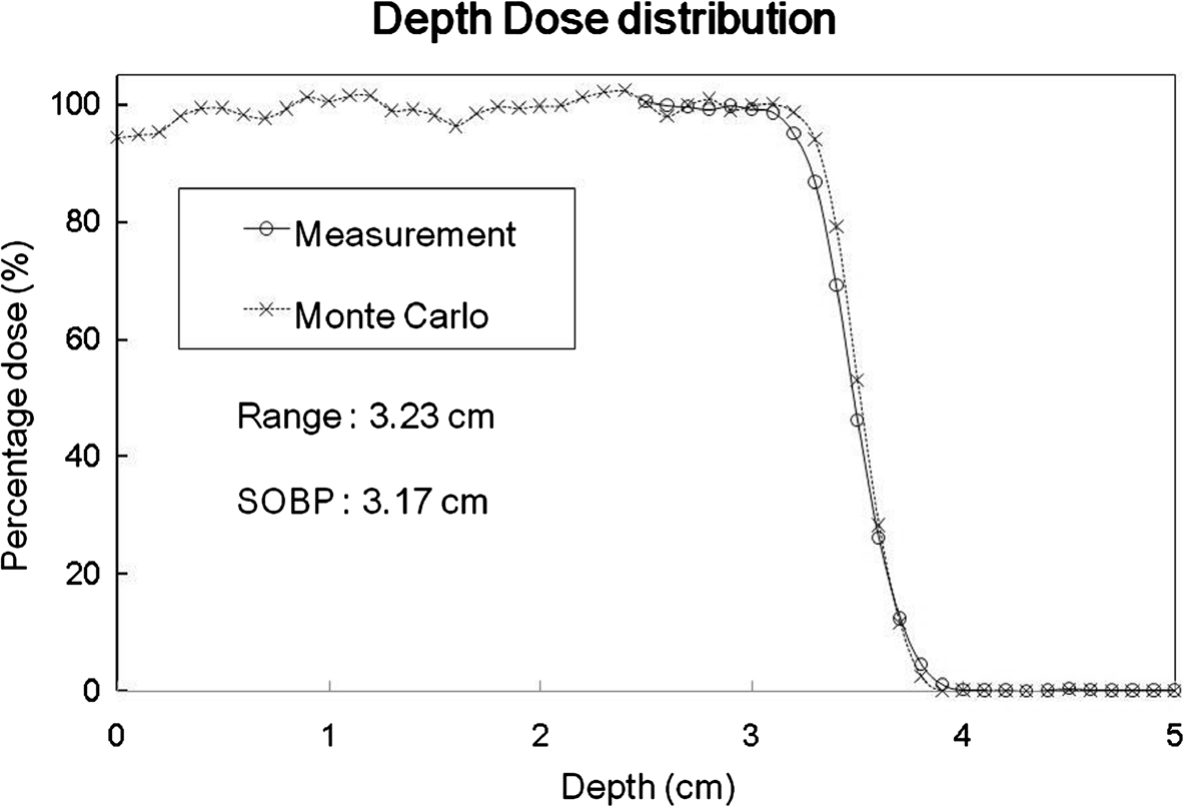
**SOBP of the proton eye beam delivery system.** Comparison of the SOBP of the proton eye beam delivery system between the measurement (circle) and Monte Carlo result (cross). The range and SOBP were 3.23 and 3.17 cm, respectively.

Figure 3 shows the percentage depth dose distributions of both the MC calculation and measurements. The range difference between the measurement and the MC calculation was 1 mm. The chamber measurement for the proton eye beam percentage depth dose was measured from a depth of 2.7 cm to account for the 1.7-cm wall thickness of the 3D water phantom and the 1-cm margin of safety in the chamber to avoid collision between the chamber and the wall of the water phantom.

## Results

### Monte Carlo calculation

**Figure 4:**
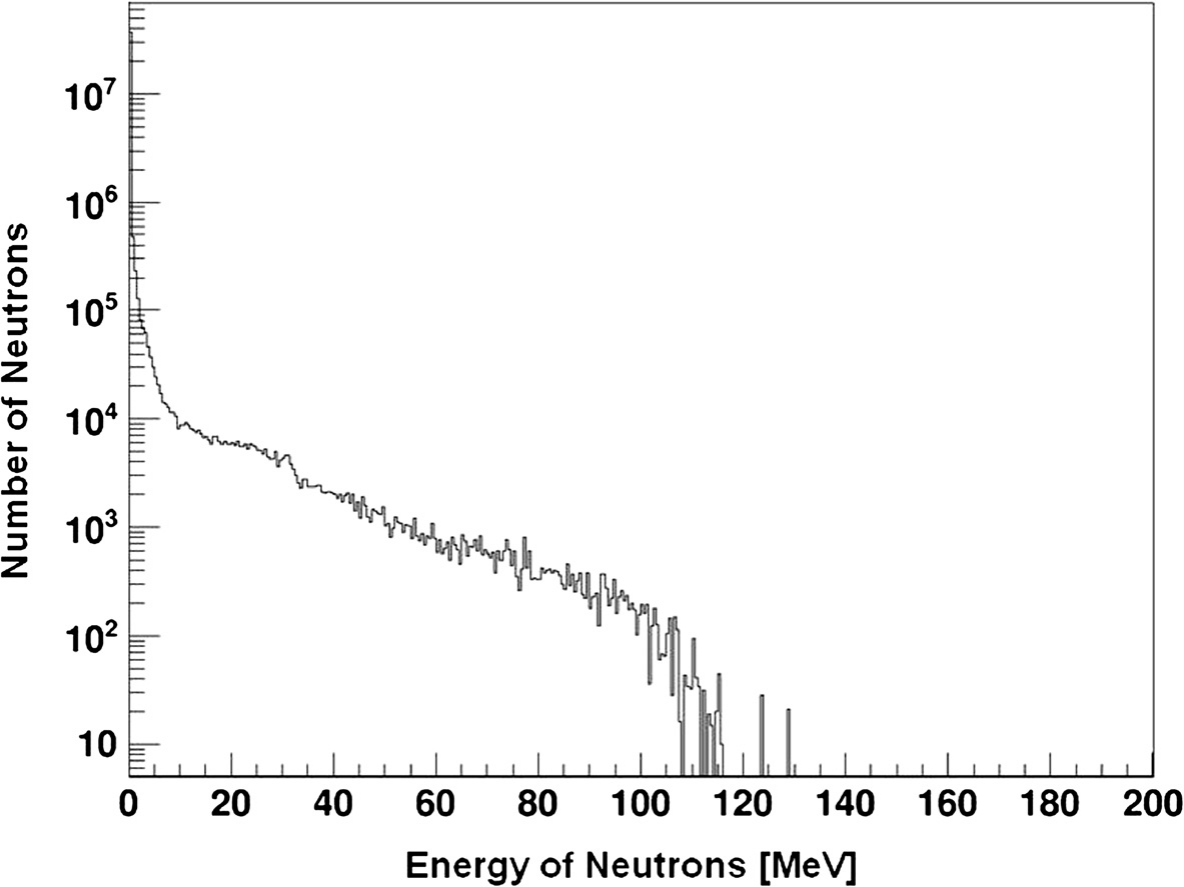
**Energy distribution of the secondary neutrons calculated by the Geant 4 Monte Carlo simulation.** Calculated neutron energy spectrum binned in 1 MeV intervals on eye proton delivery system.

**Figure 5:**
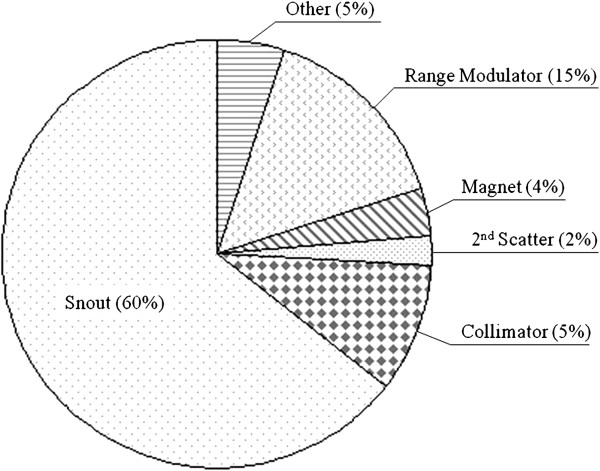
**Portions of secondary neutron generation in the beam delivery system.** View of the portions of secondary neutron generation at each sub-part inside the eye treatment beam delivery system.

Using the GEANT4 MC method, we simulated the proton eye beam delivery system at the NCC. Figure 4 shows the calculated neutron energy spectrum binned in 1 MeV intervals of the eye proton delivery system. The portion of neutrons in the total neutron energy spectrum with energies greater than 20 MeV was 2.3%. Therefore, the inefficiency caused by the limitation of the CR-39 detector for high-energy neutron detection was relatively small. In the present study, the data did not include the dose from neutrons with energies higher than 20 MeV. Figure 5 shows the portion of secondary neutrons generated at each sub-part of the eye beam delivery system when secondary neutrons from 66,797 incident protons entered the 26 × 26 × 20 cm^3^ water phantom. From the simulation study, we found that about 60% of the secondary neutrons are generated at the eye snout.

### Variation in the external field neutron dose equivalent with distance from the field edge

**Figure 6:**
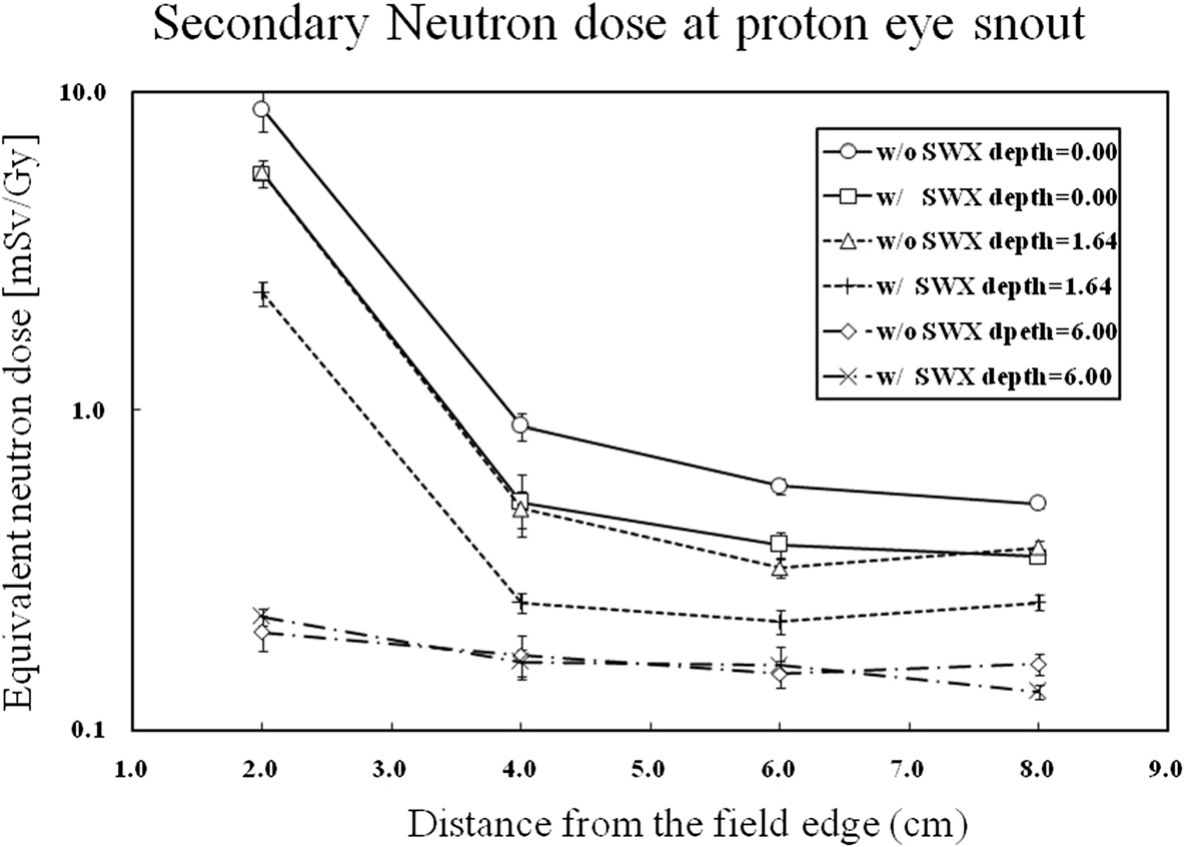
**Secondary neutron dose at the proton eye snout.** External neutron doses equivalent to the proton absorbed doses (mSv/Gy) according to the distance at the surface, in the middle of the SOBP (1.64 cm), and after the distal fall-off (60 cm) in the water phantom. Circles show the results at the surface without the SWX207HD5. Rectangles show the results at the surface with the SWX207HD. Triangles show the results at a depth of 1.64 cm without the SWX207HD5. Plus signs show the results at a depth of 1.64 cm with the SWX207HD5. Diamonds show the results at a depth of 6 cm without the SWX207HD5. Crosses show the results at a depth of 6 cm with the SWX207HD5.

Table 1 and Figure 6 show the neutron doses equivalent to the proton absorbed doses at various depths and distances from the isocenter beam. These measurements were taken outside the 4-cm-diameter primary field. The neutron dose equivalents to the proton absorbed doses (H(10)/D) (mSv/Gy) measured 2–8 cm from the primary field edge ranged from 0.51 ± 0.02 to 8.79 ± 1.28 at the surface (group A), from 0.37 ± 0.02 to 5.60 ± 0.13 at a depth of 1.64 cm (group B), and from 0.16 ± 0.01 to 0.20 ± 0.03 at a depth of 6 cm (group C). Although a previous study reported a neutron dose equivalent of 0.02 mSv/Gy with an ocular beam at distances greater than 20.0 cm [3], it is difficult to compare these results directly with ours because of differences in the measurement positions and the eye-snout systems. Nevertheless, the values were similar.

**Table 1:** External neutron dose

**Group**	**Depth [cm]**	**SWX207**	**Distance from beam edge [cm]**			
			**2.00**	**4.00**	**6.00**	**8.00**
A	0.00	W/O	8.79 ± 1.28	0.90 ± 0.08	0.58 ± 0.03	0.51 ± 0.02
		W/	5.56 ± 0.53	0.52 ± 0.11	0.38 ± 0.04	0.35 ± 0.01
		Ratio	0.63 ± 0.06	0.58 ± 0.13	0.66 ± 0.08	0.67 ± 0.02
B	1.64	W/O	5.60 ± 0.13	0.49 ± 0.06	0.32 ± 0.02	0.37 ± 0.02
		W/	2.34 ± 0.20	0.25 ± 0.02	0.22 ± 0.02	0.25 ± 0.01
		Ratio	0.42 ± 0.04	0.51 ± 0.07	0.69 ± 0.08	0.68 ± 0.03
C	6.00	W/O	0.20 ± 0.03	0.17 ± 0.03	0.15 ± 0.02	0.16 ± 0.01
		W/	0.23 ± 0.01	0.16 ± 0.02	0.16 ± 0.02	0.13 ± 0.01
		Ratio	1.15 ± 0.13	0.94 ± 0.20	1.07 ± 0.18	0.82 ± 0.06

External neutron doses (H(10)/D (mSv/Gy)) at various depths and distances from the beam isocenter with and without SWX207HD5 in single-scattering mode.

### Change in neutron dose with SWX207HD5

Table 1 and Figure 6 show the neutron doses equivalent to the proton absorbed doses at various depths and distances from the isocenter for the proton beam with and without SWX207HD5. The external field neutron doses equivalent to the proton absorbed doses (mSv/Gy) without and with SWX207HD5 at the surface of the water phantom were 8.79 ± 1.28 and 5.56 ± 0.53 at 2.00 cm from the primary field edge, respectively. The ratio of the neutron dose equivalent to the proton absorbed dose with and without a high-hydrogen–boron-containing block on the surface was 0.63 ± 0.06 at 2.00 cm from the edge of the field. The ratio of the neutron dose equivalent to the proton absorbed dose with and without a high hydrogen-boron containing block at depths of 0.00 cm, 1.64 cm, and 6.00 cm was 0.63 ± 0.06, 0.42 ± 0.04, and 1.15 ± 0.13, respectively, at 2.00 cm from the edge of the field. At a depth of 6.00 cm, the ratio of the neutron dose equivalent to the proton absorbed dose with and without a high hydrogen-boron containing block was higher than 1.0 with a large error because of the relatively small magnitude of the dose after distal fall-off of the proton beam. We found the maximum external neutron dose equivalent to the proton absorbed dose without SWX207HD5 was 60% higher than that with SWX207HD5 (Figure 6).

## Discussion

When the 2.50-cm-diameter core of the eyebeam is injected from the nozzle beam pipe into beam delivery system in the fixed-beam room, the eyebeam is spread out to 19.80-cm diameter owing to scattering by the materials in the beam path as shown as Figure 2. Subsequently, the diameter of the eye beam is reduced to 4.00 cm at the end of the eye snout. Except for the 4.00-cm opening, the proton eye beam is stopped by the eye snout material, and secondary neutrons may be produced when the eye beam passes through the ion chambers, range modulator, mirror, collimators, and/or the eye snout. Nuclear interactions between the protons and the materials in the beam path may produce secondary neutrons when proton beams are used for patient treatment or quality assurance testing.

Figure 6 shows that the external field neutron doses equivalent to the proton absorbed doses (mSv/Gy) ranged from 8.79 ± 0.16 mSv/Gy at 2.00 cm to 0.51 ± 0.02 mSv/Gy at 8.00 cm displacement from the field edge on the surface of the water phantom. These values are comparable to previously determined values of approximately 0.03 mSv/Gy at 25.00 cm and 0.02 mSv/Gy at 50.00 cm displacement from the isocenter by Massachusetts General Hospital (MGH) [3]. Recently silicon microdosimetry was used to at MGH determine that the external field neutron dose equivalent value ranges from 0.60 mSv/Gy at 2.50 cm to 0.07 mSv/Gy at 10.00 cm displacement from the field edge for a range of 2.70 cm and an SOBP beam of 2.50 cm with a 2.80 cm pre-collimation field diameter [12]. In spite of differences in the eye snout design and the collimator field size, their results are relatively consistent with our findings when comparing the large field observed in the proton double-scattering mode or by a spot beam [3,4]. It is difficult to compare these earlier results directly with ours because the neutron dose depends strongly on the material in the beam path. Thus, the results will depend on the beam delivery system, including the eye snout design. Moreover, the measurement positions used in the two studies were quite different [3,4].

To assess the neutron dose [10,11] created by the interaction between the proton beam and the patient’s body, we measured the neutron dose equivalent to the proton absorbed dose for the lateral direction in the middle of the SOBP (1.64 cm) and after the distal fall-off (6 cm). As shown in Table 1 (Group B), the neutron doses equivalent to the proton absorbed doses ranged from 5.60 ± 0.13 mSv/Gy at 2.00 cm to 0.37 ± 0.02 mSv/Gy at 8 cm displacement from the field edge. In addition, the neutron doses equivalent to the proton absorbed doses at the 6 cm position after the distal fall-off became non-negligible; 0.20 ± 0.03 mSv/Gy at 2.00 cm to 0.16 ± 0.01 mSv/Gy at 8.00 cm as shown in Table 1 (Group C).

Neutrons can be attenuated by a two-step procedure. The first step is to slow the neutrons because high-energy neutrons do not stop. This requires high-density materials in the eye beam delivery system for inelastic collisions, and hydrogenated materials to reduce the energy of the neutrons. After the neutrons are slowed, they can be captured by an absorbing material (boron) as a final step. We found that the SWX207HD5, which contains 5.50% boron and 5.70% hydrogen, can reduce the neutron dose in our proton eye beam delivery system. The neutron doses equivalent to the proton absorbed doses with and without the SXW207HD5 was separated by more than 2 times the error with an average of about 5 times the error. Moreover, the neutron dose without the SWX207HD5 was about 60% higher than the neutron dose with the SWX207HD5 when measured 2.00 cm from the displacement edge as shown in Table 1 (group A). When the depth was less than the distal fall-off, the neutron dose without the SWX207HD5 was 46–138% higher than the neutron dose with the SWX207HD5. For the neutron dose after distal fall-off, there were no significant differences between the cases with and without the SWX207HD5. It is known that CR-39 has less sensitivity to low-energy neutrons such as thermal neutrons; however, the energy weighted neutron dose equivalent [23] ranged from 5 to 50 KeV from Monte Carlo study is about 1.4% comparing to the energy weighted neutron dose equivalent for higher energy neutrons. Therefore, the less sensitivity of the CR-39 to low energy neutron will not influence to the measurement in this study.

Although, the SWX207HD5 reduced the neutron dose as described above, secondary neutrons can be generated in other places in the aperture and the patient’s body. Neutron generation in the aperture can be reduced by changing the aperture material from brass to SWX207HD5. At 2009, Brenner et al. [24] reported a reduction of the secondary neutron dose in passively scattered proton radiotherapy by using an optimized pre-collimator. In his report, the secondary neutron dose calculated using a Monte Carlo simulation for different pre-collimator materials such as the brass, tungsten alloy, Cerrobend, nickel, iron, and SWX207HD5. He reported the collimator-produced neutron dose can be reduced by using a high-density polyethylene pre-collimator in spite of the fact that it lies within the required collimator thickness (195 mm for 235-MeV protons). However, neutron generation in a patient’s body inside the region of the proton beam field is not reducible.

In 2008, Fontenot et al. [25] reported the majority of the effective dose and equivalent dose came from external neutrons created in the nozzle. They also showed that internal neutrons created in the patient contributed significantly to the equivalent doses in tissue near the proton treatment field. About 40% of the equivalent dose from neutrons was contributed by internal neutrons. In the present study, the ratio of neutron dose equivalent with SWX207HD5 to without SWX207HD5 on the surface (or middle of the SOBP) measured greater than 1, which caused by the contribution of internal neutrons to become relatively lower than the external neutrons created in the eye snout. However, the reduction of the neutron dose equivalent by neutron-shielding material such as SWX207HD5 is relatively decreased by increasing the contribution of the internal neutrons inside the phantom at greater depth. Therefore, secondary neutron generation in the patient’s body in the field region and its effect on normal tissue will be an important and interesting issue worthy of further investigation.

## Conclusions

We concluded that the secondary neutron dose for a typical eye treatment beam in the NCC, Korea, ranges from 0.16 ± 0.01 mSv/Gy to 8.79 ± 1.28 mSv/Gy with the eye snout system when the displacement from the field edge ranges from 2.00 cm to 8.00 cm. This finding indicates that the secondary neutron dose is relatively small when compared with other treatment methods. Thus, proton therapy for eye treatment will have the advantages of precise target conformity and a low integral dose with negligible secondary neutron doses. Furthermore, we found that the secondary neutron dose could be reduced by installing a neutron-absorbing material such as SWX2007HD5. However, neutron generation in a patient’s body is not reducible. Therefore, it will be very important to understand secondary neutron generation inside a patient’s body and to estimate the probability of complications in normal tissue induced by these internal neutrons. In addition, a neutron-absorbing material can be used to reduce the secondary neutron doses at other treatment sites by changing the aperture material from brass to a neutron-absorbing material, which may result in large neutron doses in craniospinal irradiation (CSI) [13,14].

## Competing interests

The authors declare that they have no competing interests.

## Authors’ contributions

DWK and DS designed and wrote a first version of the manuscript for this research. JS provided the GEANT4 Monte Carlo results. WKC, SP, and DOS provided the clinical support. YKL, SBL, JC, and MY participated in the measurements and the detector calibrations. All authors read and approved the final manuscript.
